# Antibody Engineering Using Phage Display with a Coiled-Coil Heterodimeric Fv Antibody Fragment

**DOI:** 10.1371/journal.pone.0019023

**Published:** 2011-04-28

**Authors:** Xinwei Wang, Pinyu Zhong, Peter P. Luo, Kevin C. Wang

**Affiliations:** Biologics Discovery, Merck Research Laboratories, Merck & Co., Inc., Palo Alto, California, United States of America; London School of Hygiene and Tropical Medicine, United Kingdom

## Abstract

A Fab-like antibody binding unit, ccFv, in which a pair of heterodimeric coiled-coil domains was fused to V_H_ and V_L_ for Fv stabilization, was constructed for an anti-VEGF antibody. The anti-VEGF ccFv showed the same binding affinity as scFv but significantly improved stability and phage display level. Furthermore, phage display libraries in the ccFv format were constructed for humanization and affinity maturation of the anti-VEGF antibody. A panel of V_H_ frameworks and V_H_-CDR3 variants, with a significant improvement in affinity and expressibility in both *E. coli* and yeast systems, was isolated from the ccFv phage libraries. These results demonstrate the potential application of the ccFv antibody format in antibody engineering.

## Introduction

The field of therapeutic antibody development has exploded in the past two decades with the invention of various antibody formats [1], [2]. These novel antibody fragment formats are possible because antigen recognition occurs through a discrete region of the molecule known as the Fv fragment. Comprised of two variable regions, the heavy and light chains (V_H_ and V_L_, respectively), the Fv fragment contains all the structural information necessary for high affinity antigen binding.

Although the Fv fragment can recognize an antigen, the interaction energy between the V_H_ and V_L_ chains is low. Thus, Fvs are often too unstable for therapeutic applications. In naturally occurring immunoglobulins (Igs), the V_H_ and V_L_ regions are held together by the C_H_1 and C_L_ domains as well as by an interchain disulfide bond. These four regions, together with the interchain disulfide bond, comprise a Fab. In contrast to the smaller Fv fragment, Fabs are much more stable, making them more broadly applicable not only to research but also to immunodiagnostic and immunotherapeutic applications; however, this stability results from a relatively high molecular weight of 50 kDa.

The ideal antibody fragment would have three properties: small size, high binding affinity (nanomolar or below), and high stability, including the ability to withstand physiological salt, pH and temperature conditions. In light of these requirements, a variety of approaches have been taken to build a functional, stable complex of V_H_ and V_L_. The commonly used single chain Fv, or “scFv,” approach utilizes a flexible peptide linker to covalently connect the V_H_ and V_L_ domains into a single polypeptide [3], [4]. The resulting scFv exhibits substantial antigen-binding activity compared to a non-disulfide-bonded Fv fragment. However, scFvs tend to form dimers and aggregates [5], [6] and are relatively unstable compared to Fabs [7]. scFv molecules need to be further engineered to improve their stability for many therapeutic applications.

We and others have created Fab-like molecules (hsFv and ccFv) by replacing the C_H_1 and C_L_ domains with smaller heterodimeric domains. The hsFv complex utilized two engineered helical domains from Fos and Jun to dimerize the V_H_ and V_L_ domains from an anti-phosphorylcholine antibody, yielding a helix-stabilized Fv fragment [8]. In the ccFv construct, the V_H_ and V_L_ domains were dimerized via a pair of naturally occurring coiled-coil domains derived from the heterodimeric GABA_B_ receptors [9]. These Fab-like formats confer the desired advantages of relatively small size (approximately 35 to 37 kDa) and high stability and affinity.

Here, we report the characterization of an anti-VEGF ccFv antibody. Our data showed that the ccFv molecule had a binding affinity similar to the corresponding scFv but was significantly improved in thermostability and phage display level. Furthermore, ccFv phage display libraries were constructed for humanization and affinity maturation of the anti-VEGF antibody. A panel of V_H_ frameworks and V_H_-CDR3 variants with significant improvements in affinity and expressibility were isolated by library panning. This study demonstrates the utility of the ccFv format in antibody engineering and its feasibility for diagnostic and immunotherapeutic applications.

## Results

### ccFv construct

**Figure 1:**
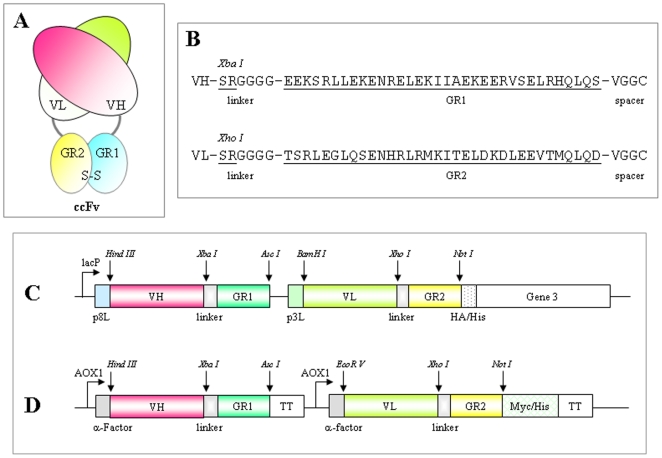
An overview of the coiled-coil Fv (ccFv) construct. (A) The ccFv molecule design. The V_H_ and V_L_ are heterodimerized through the interaction of GR1 and GR2 coiled-coil domains. (B) The amino acid sequences of GR1, GR2, linker, and spacer. (C) pABMD5, the ccFv phage display vector. (D) pABMX168, the ccFv expression vector in *Pichia*.

To construct a Fab-like molecule (Figure 1A), it was necessary to select an optimal pair of heterodimerization domains to fuse with V_H_ and V_L_. Based on several desirable features, we chose the coiled-coil domains GR1 and GR2, at 4 KDa each approximately, from the GABA_B_-R1 and GABA_B_-R2 receptors. A previous study has demonstrated that the GR1 and GR2 peptides preferentially form parallel coiled-coil heterodimers under physiological buffer and temperature conditions. The GR1 coiled-coil peptides folded into relatively unstable homodimers, whereas the GR2 coiled-coil peptides were largely unstructured and could not form homodimers [10].

To provide additional flexibility for the heterodimerization of V_H_ and V_L_, we modified the amino termini of GR1 and GR2 by adding a 6-amino-acid flexon (Ser-Arg-Gly-Gly-Gly-Gly). To further stabilize the ccFv construct, a pair of cysteine residues was added via a spacer (Val-Gly-Gly-Cys) at the C-termini of the GR1 and GR2 domains. These domains are, in turn, fused to the carboxyl termini of the V_H_ and V_L_ fragments, respectively. The sequences of GR1 and GR2 with the above modifications are listed in Figure 1B. Because the antibody V_H_ and V_L_ domains are heterodimerized through the coiled-coil domains, the new format is named a coiled-coil Fv or “ccFv”.

### ccFv expression and stability

The V_H_ and V_L_ sequences of a humanized anti-VEGF antibody [11] were selected and converted to anti-VEGF ccFv and scFv (a control). To characterize the newly created ccFv, the anti-VEGF ccFv and scFv were first cloned into a bacterial vector for expression. However, both ccFv and scFv had a low expression yield (below 20 µg/L). Therefore, we switched to a *Pichia* system to express the anti-VEGF ccFv and scFv antibodies; the expression vector for ccFv is shown in Figure 1D. The *Pichia* system significantly improved the expression yield. A total of 50 ml induced culture generated approximately 0.5 to 1 mg of purified ccFv and scFv antibody proteins, which were enough for further characterization.

**Figure 2:**
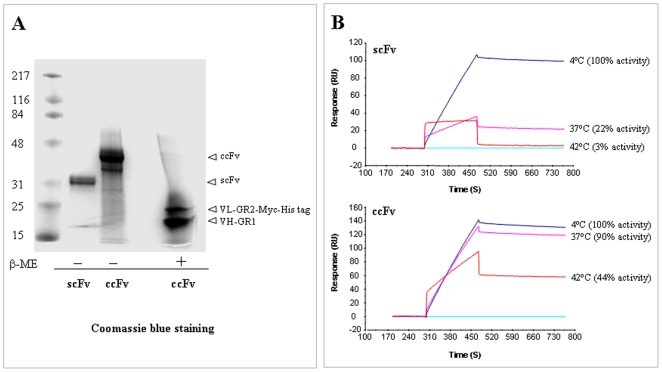
The expression of the ccFv fragment in *Pichia* and the study of its stability. (A) SDS-PAGE analysis of purified ccFv and scFv, under reducing (β-ME) and non-reducing conditions. ccFv showed an electrophoretic mobility of approximately 35 kDa. Under reducing conditions, two ccFv subunits corresponding to V_L_-GR2-Myc-His tag and V_H_-GR1 were observed. (B) A thermal stability comparison between ccFv and the corresponding scFv. The anti-VEGF ccFv and scFv fragments were incubated at 4°C (referred to as 100% binding activity), 37°C, and 42°C. Their remaining binding activity to the VEGF antigen was measured by Biacore at 25°C.

The *Pichia*-expressed soluble anti-VEGF ccFv and scFv antibody fragments were subsequently purified with a Ni-NTA column via their histidine tags. Figure 2A shows that the purified ccFv has an electrophoretic mobility of approximately 35 kDa on a non-reducing gel. Under reducing conditions, two subunits corresponding to V_L_ and V_H_ were observed. Western blotting with an anti-Myc-tag antibody confirmed the identity of the upper band as the V_L_-Myc-His-tag fusion protein (data not shown). Furthermore, the Biacore measurement showed that the ccFv bound to the VEGF antigen with 10.3 nM affinity, which was similar to that of scFv (13.8 nM). These results demonstrated that the ccFv molecule could be successfully expressed and functionally assembled in *Pichia* cells.

The stability of the anti-VEGF ccFv was compared to that of its corresponding scFv antibody. The bindings of the antibodies to VEGF were measured at 25°C after a 16 h incubation at either 4°C, 37°C or 42°C. Their remaining ability to bind to the antigen was measured by Biacore (Figure 2B). The ccFv antibody exhibited significantly higher stability than the scFv antibody, with 90% and 44% of antigen binding activities remaining after 37°C and 42°C overnight incubations, respectively, whereas only 22% and 3% of the antigen binding activity of the scFv remained after the same treatments.

Taken together, these results indicate that the novel antibody fragment construct ccFv showed affinity and expressibility comparable to scFv and better stability than scFv.

### Use of ccFv in phage display

**Figure 3:**
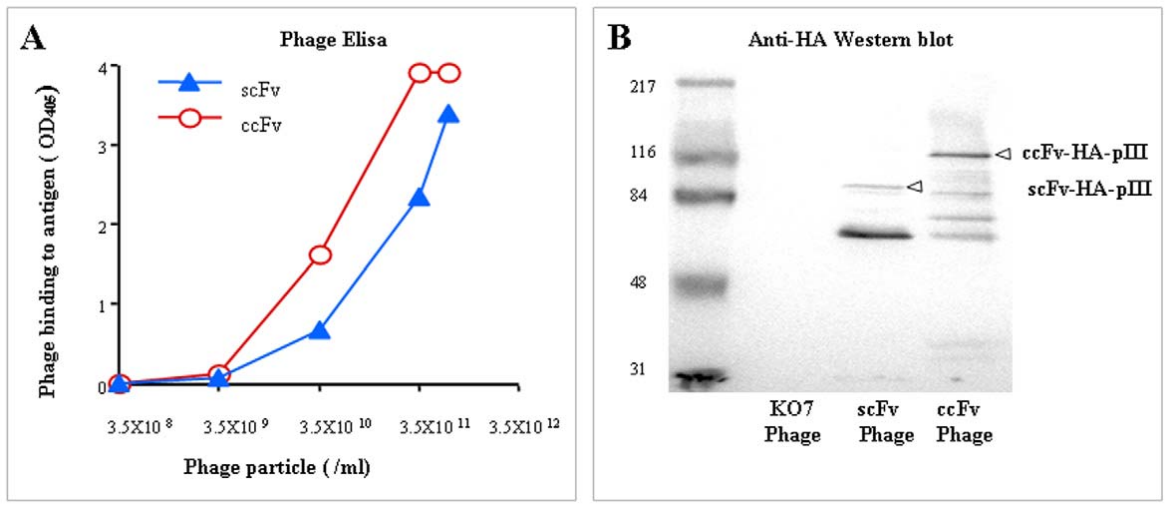
The functional phage display of the ccFv fragment. (A) Phage ELISA was conducted using the phages displaying either the ccFv or scFv fragment encoding the same anti-VEGF antibody. The results demonstrated that ccFv was functionally assembled, expressed, and displayed on phages. The display level was higher by almost one order of magnitude than scFv. (B) A western blot of phages displaying ccFv and scFv fragments. The same amounts of phage particles displaying either the ccFv or scFv fragment were analyzed by SDS-PAGE and anti-HA western blotting. More intact ccFv-pIII fusion proteins were detected than the intact scFv-pIII fusion proteins, suggesting a higher ccFv phage display level.

To demonstrate the utility of ccFv in a phage display system, we constructed a phagemid vector by subcloning the anti-VEGF-ccFv gene into the pABMD5 vector (Figure 1C). To compare the ccFv to the scFv in phage display, phagemids encoding both the ccFv and scFv anti-VEGF fragments were prepared. As shown in Figure 3A, phages displaying either the ccFv or scFv bound specifically to the antigen VEGF in a dose-dependent manner. The positive ELISA signal indicated the successful assembly and expression of the functional ccFv fragments on phages. Moreover, the phages displaying the ccFv fragments exceeded those displaying the scFv by almost one order of magnitude in binding.

To verify the display levels, the same amounts of phage particles displaying either ccFv or scFv were subjected to SDS-PAGE and anti-HA-tag western blotting. As shown in Figure 3B, more intact ccFv-pIII fusion proteins were observed than the intact scFv-pIII, suggesting a higher ccFv display level, which was consistent with the ELISA results.

To demonstrate that a specific ccFv phage could be selected from a ccFv phage display library, we constructed a “doped library” and panned for specific binders. Phages displaying an anti-VEGF ccFv fragment were mixed with control phages displaying ccFv fragments that do not bind to VEGF in a ratio of 1∶10^7^. A total of 10^12^ phages were used in two rounds of panning against immobilized VEGF. After each round of panning, the bound library phages were harvested and tested for antigen recognition in an ELISA against immobilized VEGF. Simultaneously, to monitor the actual enrichment of the panning, the ratio of the anti-VEGF phage to the background phages was measured. Randomly picked clones were analyzed by PCR via a pair of primers specifically designed to amplify only the anti-VEGF ccFv and generate a band of approximately 1 kb. The phages from the second round generated an ELISA signal of OD_405_ 3.8, significantly higher than the signal of OD_405_ 0.05 from the first round, demonstrating that the anti-VEGF-ccFv-displaying phages could be successfully selected by panning. PCR analysis confirmed the enrichment of the anti-VEGF ccFv. Although the original 1∶10^7^ doped library contained only 0.00001% anti-VEGF ccFv, its presence increased to 4% (2/25) after a single round of panning and reached nearly 100% (14/14) after the second round.

### Application of ccFv in antibody humanization

**Figure 4:**
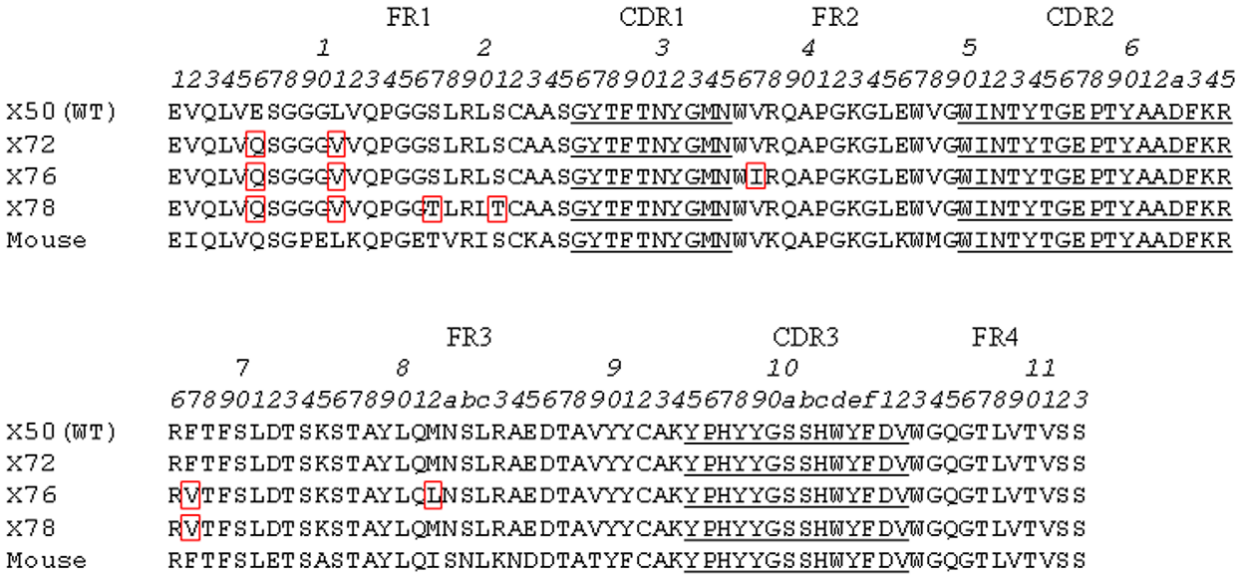
The framework amino acid sequences of the leads selected from the ccFv humanization library for the anti-VEGF antibody. All three leads, X72, X76, and X78, differed from WT at the E6Q and L11V mutations in the V_H_ framework 1. The Kabat numbering scheme was used for residue numbering.

To examine the utility of the ccFv format in antibody engineering, a V_H_ library was constructed in the ccFv format to humanize a murine anti-VEGF antibody [12]. This library, with a diversity of 1.6×10^6^, was designed based on V_H_ structure modeling and the human germline sequence. The ccFv library phages were used to select against human VEGF. After 8 rounds of panning, three dominant hits, X72, X76, and X78, were identified. X72 represented 50% of the ELISA-positive clones, X76 25%, and X78 5%. The framework sequences of X72, X76, and X78 are shown in Figure 4. Phylogenic analysis indicates that these V_H_ frameworks are undistinguishable from human germline families.

Compared to the previously humanized antibody V_H_ sequence (referred to as WT) [11], different framework residues were identified in all three hits. The E6Q and L11V changes in FR1 were shared by all three hits. X76 also differed from the WT at one position in FR2 and at two positions in FR3, whereas X78 differed at two more positions in FR1 and one position in FR3 (Fig. 4).

**Figure 5:**
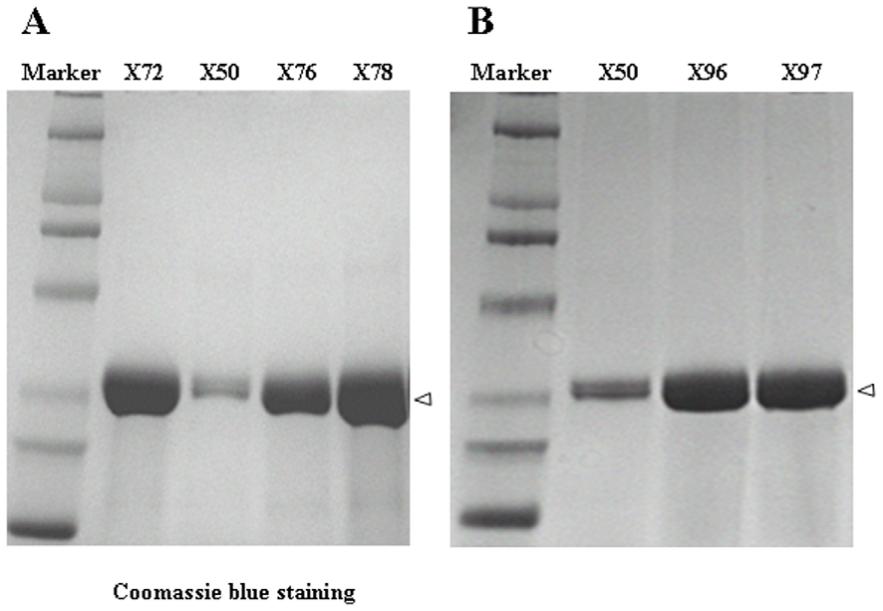
Improved expression of the leads identified from the ccFv humanization and maturation libraries for an anti-VEGF antibody. The leads identified from the ccFv libraries were converted to scFv and expressed in *Pichia*. (A) SDS-PAGE analysis of the expression of the humanization leads (X72, X76, and X78) and the WT control. All three leads showed an expression level significantly higher than that of WT X50. (B) SDS-PAGE analysis of the expression of X96 and X97, which carried the combined mutations identified from both the humanization and the maturation libraries.

The expressibility and affinity of the three hits were further characterized. To exclude any potential bias from the new ccFv format, the hits and the WT were converted to the established scFv format for *Pichia* expression. The his-tagged scFv fragments were purified by a Ni-NTA column from methanol-induced culture supernatants and analyzed by SDS-PAGE. As shown in Fig. 5A, the expression levels of X72, X76, and X78 were significantly higher than that of the WT X50. With experimental variation from batch to batch, the expression yields of all three hits were 5- to 10-fold higher than that of the WT. In addition, Biacore measurements indicated that the affinities of the new hits increased 2.3- to 4.5-fold relative to the WT (Table 1).

**Table 1:** Affinity of variants post humanization or maturation at 25°C.

Hit	*k*_on_×10^3^	*k*_off_×10^−4^	K_d_ (nM)	K_d_^WT^/K_d_^Hit^
WT	6.14±0.04	0.85±0.01	13.8	1
X72	17.9±0.13	0.69±0.05	3.9	3.5
X76	37.4±0.50	1.15±0.01	3.1	4.5
X78	12.2±0.09	0.72±0.05	5.9	2.3
X63	1.14±0.06	0.025±0.02	2.2	6.3
X64	15.9±0.09	0.867±0.03	5.4	2.5
X65	2.25±0.04	0.034±0.01	1.5	9.2

### Application of ccFv in antibody affinity maturation

To improve the affinity of the anti-VEGF antibody, two combinatorial CDR3 libraries in the ccFv format, Libraries 1 and 2, were constructed in the WT framework. Each phage library was separately panned against immobilized VEGF. After multiple rounds of panning, 92 clones were randomly picked from each library and examined by phage ELISA. All clones demonstrated positive binding activity to VEGF.

Library 1 had diversity at positions 95, 97, 99 to 100c and 102. After 5 rounds of panning, the mutations at positions 97 and 100a were identified. All positive clones carried the 97 histidine (H) to tyrosine (Y) change, and 80% of clones had a serine (S) to threonine (T) mutation at position 100a. From Library 2, with diversity at positions 94 to 96, 100a to 100d, and 102, after 7 rounds of panning, 70% of clones had the change from 100a serine (S) to arginine (R), whereas 30% had the serine (S) to threonine (T) change that was dominant in the Library 1 selection. These mutations have also been reported in a previous study [13].

Three clones, X63 (with the mutations H97Y and S100aT), X64 (with the mutation S100aR), and X65 (with the mutations H97Y and S100aR), were converted to scFv formats and expressed in *Pichia*. Their affinities were measured by Biacore and showed 2.5- to 9.2-fold improvement than the WT (Table 1).

Next, the V_H_-CDR3 mutants in X65 were grafted into X72 and X76 frameworks, resulting in two constructs, X96 and X97, respectively. The scFv formats of these two variants were then expressed in *Pichia* to examine their expressibility. As shown in Figure 5B, both constructs maintained the good expressibility inherited from the X72 and X76 frameworks. Their affinities were determined by Biacore analysis at the elevated temperature of 35°C due to the slow dissociation rate at 25°C. The results in Table 2 show that both X96 and X97 exhibited a greater than 30-fold improvement in VEGF binding affinity.

**Table 2:** Affinity of VH variants at 35°C.

Hit	*k*_on_×10^4^	*k*_off_×10^−4^	K_d_ (nM)	K_d_^WT^/K_d_^Hit^
WT	7.6±0.03	2.90±0.01	3.8	1
X96	8.5±0.04	0.09±0.01	0.11	35
X97	11.5±0.1	0.14±0.03	0.12	31

Furthermore, the expressibility of the improved clones X96 and X97 in the Fab format was examined in *E. coli*. The Fabs were expressed in TG1 cells and purified from periplasm by protein G columns. Consistent with the improvement in *Pichia*, a significant improvement in Fab expression in *E. coli* was observed as well. The purification yields of the X96 and X97 Fabs reached 229 and 303 µg per liter in TG1 culture, respectively, approximately 10- and 15-fold higher than that of the WT Fab, which was 21 µg/L.

## Discussion

The coiled-coil leucine zipper domains have been used to reconstitute a variety of heterodimeric complexes [14]. With the discovery of more leucine zipper sequences and growing insights into the mechanism of helix formation, several groups have pursued library approaches to engineer interacting domains that might best induce heterodimer assembly [15], [16]. In such libraries, the key positions of the helical structure were partially randomized and the best interacting partners were selected through “library against library” screening. Both engineered and naturally occurring coiled-coil domains have been utilized in the efforts to construct the smaller Fab-like molecules [8], [9].

WinZip-A2B1, an engineered heterodimeric coiled-coil pair, was selected simultaneously from two libraries using an *in vivo* protein-fragment complementation assay with dihydrofolate reductase [16]. This selected peptide pair greatly favored heterodimerization over homodimerization. However, similar to the parental Jun/Fos leucine zipper pair, both WinZip-A1 and WinZip-B1 can form homodimers, whereas the homodimeric WinZip-A1 is almost as stable as the heterodimer. Because this leucine zipper pair was overwhelmingly selected using *E. coli*, it was assumed that the helical domains were metabolically stable and functionally active in such system. The WinZip-A2B1 coiled-coil domains were used to heterodimerize the V_H_ and V_L_ of an anti-phosphorylcholine antibody, creating the helix-stabilized Fv fragment hsFv [8]. In contrast to the scFv format, which is prone to aggregation, the hsFv format showed higher stability and no tendency to oligomerize or aggregate. The hsFv also boasts a relatively small size of 37 kDa.

In this study, we designed a functional antibody fragment format, the ccFv, by inducing the dimerization of a pair of naturally occurring, intracellular coiled-coil domains. Fos and Jun, the well-characterized coiled-coil leucine zipper proteins, can heterodimerize but are prone to homodimerization as well [17]. In contrast, the C-terminal coiled-coil domains (GR1 and GR2) of the GABA_B_-R1 and GABA_B_-R2 receptors do not form detectable homodimers either *in vivo* or *in vitro.* Previous studies have demonstrated the heterodimerization specificity of GR1 and GR2 *in vivo* [18], [19]. In fact, White *et al*. [19] have been able to clone GR2 from a yeast two-hybrid system based on the exclusive specificity of this heterodimeric receptor pair. *In vitro* studies by Kammerer *et al*. [10] have shown that neither GABA_B_-R1 nor GABA_B_-R2 C-terminal sequence can form homodimers in physiological buffer conditions at physiological body temperatures.

The current study demonstrated that the GR1 and GR2 coiled-coil domains effectively stabilize V_H_ and V_L_ in the construction of a coiled-coil Fv domain, or “ccFv”.The ccFv molecule has a shorter linker of six amino acids between the antibody variable sequences and the coiled-coil domains compared to the 13 amino acid-linker in hsFv. By using a linker of only 6 amino acids, we constructed multiple functional ccFv antibodies (data not shown), suggesting that shorter linkers worked well. Additionally, a covalent interchain bond was engineered at the C-termini of the GR1 and GR2 domains to further stabilize the ccFv construct. The ccFv antibody format was based upon previous efforts to construct a small antibody fragment that could remain stable under physiological conditions. Because of their small sizes, ccFv and its derivatives are potentially more useful than traditional antibodies for clinical applications requiring tumor and tissue penetration.

A main effort in current antibody engineering involves the use of phage display library construction and panning to select high affinity antibodies for research and therapeutic purposes [20], [21]. Panning of first a “doped” library and then the optimized libraries revealed that high-affinity, high-specificity binders can be selected using the ccFv format. In addition to the affinity improvement, we identified three humanized V_H_ frameworks that significantly improved the production of the anti-VEGF antibody in both the scFv and Fab formats. Compared to the previous humanized sequence, all three new hits shared two key mutations of E6Q and L11V in the V_H_ framework 1. Recent studies have suggested that the V_H_ framework residue at position 6 plays a critical role in antibody folding, antigen-binding activity, and determination of the local conformation of the framework 1 region [22], [23], [24]. From a structural point of view, the burial of a charged glutamate (E) residue at position 6 would be energetically unstable. It has been reported in a bacterial system that the substitution of glutamate (E) with glutamine (Q) at position 6 dramatically improved (by 30-fold) the scFv production for an anti-CD3 antibody [25]. Our data showed that the exchanges of E6Q and L11V significantly improved the expression not only in the prokaryotic *E. coli* system but also in the eukaryotic yeast (*Pichia)* system. The influence of the L11V exchange remains to be determined. Structure modeling and analysis suggested that this L11V change would be energetically favorable (data not shown).

In summary, this study demonstrated the utility of the GR1 and GR2 coiled-coil domains in reconstituting the characterized Fv fragment; the resulting coiled-coil Fv antibody fragments (ccFvs) can be effectively used in constructing large libraries and selecting novel antibodies via phage display.

## Materials and Methods

### Materials

*Escherichia coli* TG1 cells [*supE thi-1* Δ (*lac-proAB*) Δ (*mcrB-hsdSM*)*5* (r_K_^−^ mK^−^) [F' *traD36 proAB lacI* ^q^ *Z*Δ*M15*] were used for the production of plasmid DNA and phages; the KO7 helper phage and HRP-conjugated anti-M13 antibody were from Amersham Pharmacia Biotech; pBluescript SK(+) was from Stratagene; the *Pichia pastoris* expression system, including the pPIC6 alpha vector and X33 train, was from Invitrogen.

Recombinant VEGF_121_ was from Calbiochem; the HRP substrate ABTS [2,2′ Azino-bis(3-ethylbenzthiazoline-6-sulfonic acid)] and triethylamine were from Sigma; Maxisorb 96-well plates were from Nalgene-Nunc.

### Vector construction

The vectors for antibody expression and display in TG1 cells were derived from pBluescript SK(+). A unique AgeI restriction site was introduced immediately after the 3′ of the *lac* promoter by PCR-based site-directed mutagenesis with a set of primers (pBS-SKa: 5′-GGAATTGTGAGCGGATAACAATTTACCGGTCACACAGGAAACAGCTATGAC CATG-3′ and pBS-SKb: 5′CATGGTCATAGCTGTTTCCTGTGTGACCGGTAAATTGTTATCCGCTCACAATTCC-3′); the XhoI and KpnI sites were then deleted by restriction digestion and blunt end ligation. For ccFv expression, the synthetic DNA fragment flanked by an AgeI site at the 5′ end and BglII/EcoRI sites at the 3′ end and containing ribosome binding sequence-fd phage gene8 leader sequence-V_H_-GR1 coding sequences and ribosome binding sequence-fd phage gene3 leader sequence-V_L_-GR2 coding sequence-HA/His tag sequences was cloned into the modified pBluescript SK(+). The resulting vector was designated pABMX5. To display ccFv, a PCR-amplified fd gene3 fragment flanked by BglII and SalI sites was inserted into vector pABMX5 to make the phagemid display vector pABMD5 (see Figure 1C). The V_H_ and V_L_ genes of anti-VEGF antibodies A4.6.1 and Fab-12 [11] were obtained by PCR assembly of overlapping oligonucleotides, and the sequence was confirmed by DNA sequencing.

To express scFv in *Pichia*, the scFv gene was cloned into the *Pichia* expression vector pPIC6 alpha (Invitrogen) via the XhoI and NotI sites, in frame with the α-factor and Myc-His tag sequences. The ccFv *Pichia* expression vector was also derived from the pPIC6 alpha vector. The HindIII site in the 3′ end of the AOX1 promoter was eliminated by single nucleotide substitution (AAGCTT changed to AATCTT) and then inserted after the α-factor sequence. To construct the anti-VEGF ccFv *Pichia* vector, a synthetic fragment containing the V_H_-GR1 coding sequence- termination sequence AOX1TT and the AOX1 promoter-VK-GR2 coding sequence was cloned into the Hind III and Not I sites of the newly engineered pPIC6 alpha vector; the resulting construct was in frame with the α-factor and the Myc-His tag (pABMX168 in Fugure1D).

For Fab expression in *E. coli*, the V_H_ and V_K_ sequences were cloned into the modified pABMX492 vector, avoiding the GR1 sequence [26].

### Antibody expression and purification

*Pichia* X33 cells containing either ccFv or scFv constructs were grown at 30°C in 500 ml BMGY medium (100 mmol/L potassium phosphate (pH 6.0), 1.34% yeast nitrogen base with ammonium sulfate, 0.4 mg/L of biotin, 10 g/L yeast extract, 20 g/L peptone, and 1% glycerol)/kanamycin (70 mg/L) until the OD at 600 nM reached 2.5. The cells were pelleted and resuspended to a final volume of 50 ml in BMMY medium (0.5% (v/v) methanol instead of glycerol in BMGY)/kanamycin (70 mg/L)for a 5-day induction at 30°C. Methanol (0.5 ml) was added daily to maintain the methanol concentration at 1%. After methanol induction, the cells were removed by centrifugation, and the supernatant was subjected to a 1 ml Ni-NTA column (Qiagen). The bound proteins were eluted with 500 mM imidazole in PBS. Fractions of 0.5 ml were collected from the beginning of the elution, and the fractions (number 2 and 3) containing the antibody protein were combined and dialyzed against PBS. Twenty microliters of each purified sample was analyzed by SDS-PAGE.

For bacterial expression of the antibody fragments, TG1 cells harboring phagemid vectors were grown in 500 ml 2xYT medium containing 100 µg/ml carbenicillin and 0.1% glucose until the OD_600_ reached 0.7. IPTG (1 mM) was added for a 4-h induction at 30°C. After the periplasmic and osmotic shock preparation of the TG1 cell pellets, the supernatant was dialyzed against PBS and subjected to column purification. For the His-tagged scFv and ccFv, the samples were loaded into a 1-ml Ni-NTA column (Qiagen). The bound proteins were eluted with 500 mM imidazole in PBS. For Fab, the samples were loaded onto a 1-ml Protein G column (GE), and the column-bound Fabs were eluted by pH 3.0 100 mM glycine. The fractions containing antibody proteins were combined and dialyzed against PBS. Twenty microliters of each purified sample was analyzed by SDS-PAGE.

### Antibody library construction

A humanization library of size 1.6×10^6^ clones was designed for the murine anti-VEGF antibody A4.6.1 [12], [27], based on antibody structure modeling and the human germline sequence. During the operation of humanization *in silico* [28], 34 positions in the V_H_ framework were designed: 16 had designed diversity, and 18 were fixed. The library was constructed in the ccFv format. For affinity maturation, two combinatorial CDR3 libraries in the ccFv format, Libraries 1 and 2, were constructed in the humanized framework [11]. Library 1, with a size of 6.6×10^5^, had diversity at positions 95, 97, 99 to 100c and 102; Library 2, with a size of 1.7×10^5^, covered positions 94 to 96, 100a to 100d, and 102.

The humanization and maturation libraries in this study were constructed by PCR assembly of overlapping oligonucleotides containing variable nucleotides at key positions to generate mutations at specific sites. The PCR assembly was performed as follows: the library oligonucleotides were mixed to a final concentration of each oligonucleotide of 0.5 µM. Three panels of assembly reactions were set up with 0.2 mM of each dNTP, 1 mM magnesium sulfate, 5 U of Pfu Turbo DNA polymerase, and differing amounts of oligonucleotide mix (1 µl, 2 µl, and 3 µl, respectively) in 1 X Pfu buffer to a total volume of 50 µl. A total of 25 cycles of PCR (94°C 45 sec; 51°C, 45 sec; 72°C, 1 min) were performed to assemble the overlapping oligonucleotides into antibody genes. Following assembly, the antibody genes were amplified by 35 cycles of PCR (same as above) using the assembly reactions as templates. The PCR-amplified DNA fragments were then digested and cloned into their corresponding positions in the ccFv vector.

### Phage display and panning

The phagemid vector or library DNA was transformed into TG1 cells. The transformed cells were superinfected with KO7 helper phage, with a multiplicity of infection (MOI) of 5 to 10. After an overnight culture at 30°C in 2xYT medium with 100 µg/ml carbenicillin and 35 µg/ml kanamycin, the phage particles were purified by two times of PEG precipitation from culture supernatants and titered by OD_268_ measurement. The antibody displayed on the phage surface was detected by phage ELISA using plates coated with VEGF or by phage western blot.

For phage library panning, recombinant VEGF was immobilized onto Nunc Maxisorb 96-well plates in 0.05 M NaHCO_3_, pH 9.6 buffer at 4°C overnight. An aliquot of the phage library containing 10^12^ phage particles diluted in PBS buffer with 2% milk was added to the wells coated with the antigen. After incubating at room temperature for 2 h, the wells were washed with PBST (0.05% Tween-20 in PBS) and PBS. Bound phages were eluted with 100 mM triethylamine and added to a TG1 culture (grown at 37°C until the OD_600_ reached approximately 0.5 to 1.0) for infection at 37°C for 1 h followed by KO7 helper phage rescue. The rescued TG1 cells were then grown in 2xYT/carbenicillin/kanamycin at 30°C overnight. Supernatants of the bacterial cultures were harvested. The phagemid particles were precipitated by PEG/NaCl from the supernatants and resuspended in PBS for the next round of panning or phage ELISA.

### Phage ELISA

Recombinant VEGF was dissolved in 0.05 M NaHCO_3_ buffer pH 9.6 and immobilized at 1 µg/100 µl per well on ELISA plates at 4°C overnight. After blocking with 5% milk in PBS to minimize nonspecific binding, a 100 µl aliquot containing the phages to be tested was added to each well in the ELISA plate and incubated for 2 h at room temperature. The unbound phages were then washed away with PBS. The phage bound to the antigen was detected by incubation with 100 µl of HRP-conjugated anti-M13 antibody (at a 1∶1000 dilution in PBS). After a brief wash with PBS, 100 µl of ABTS (2, 2′ azino-bis[3-ethylbenzthiazoline-6-sulfonic acid]) was added before HRP activity was measured by OD_405_.

### Western blot

For each phage sample preparation, approximately 4×10^11^ phage particles were heated for 10 min in the SDS sample buffer (2% SDS, 10% glycerol, 0.67 M Tris-HCl, pH 6.8). The denatured phage samples were subjected to 10% SDS-PAGE. The proteins in the SDS gel were then transferred to a PVDF membrane. To detect HA-tagged pIII fusion proteins, the membrane was probed first with mouse monoclonal antibody HA probe F-7 (Santa Cruz Biotech) at a 1∶200 dilution in 5% MPBS and then detected by anti-mouse antibody-AP conjugate and BCIP/NBT AP substrate (Sigma).

To detect myc-tagged proteins, the membrane was probed first with 2 µg/ml mouse monoclonal antibody 9E10 in 5% MPBS and then detected by anti-mouse antibody-AP conjugate and the BCIP/NBT AP substrate (Sigma).

### Antibody activity and affinity measured with Biacore

The recombinant human VEGF (R&D Systems, 293-VE/CF) was diluted to 20 µg/ml in pH 6.5 10 mM sodium acetate and immobilized on a CM5 chip using the Amine Coupling kit (GE, BR-1000-50) according to the manufacturer's instructions. The final VEGF immobilization level was approximately 300 RU. For the thermostability study, the antibody samples were injected at 30 µl/min for a 2-min association, followed by a 5 min dissociation at the same flow rate. The binding RUs were recorded at 5 sec after initiation of dissociation. The surface was regenerated by a 30-sec injection of 10 mM, pH 1.5 glycine-HCl at 50 µl/min.

For scFv affinity measurement, the purified scFv proteins were loaded onto a Superdex 75 size-exclusion column to remove potential dimers and aggregates. The monomeric scFv proteins were diluted in PBS, and scFv samples at 5 different concentrations, 10 nM, 20 nM, 40 nM, 80 nM, and 160 nM, were injected at 30 µl/min for 5 min, followed by a 10-min dissociation at the same flow rate. The binding kinetics and affinities were determined by globally fitting the curves of 5 concentrations to a 1∶1 Langmuir model.
